# Cloning of Porcine Pituitary Tumor Transforming Gene 1 and Its Expression in Porcine Oocytes and Embryos

**DOI:** 10.1371/journal.pone.0153189

**Published:** 2016-04-08

**Authors:** Bingkun Xie, Zhaoxian Qin, Shuai Liu, Suqun Nong, Qingyan Ma, Baojian Chen, Mingjun Liu, Tianbiao Pan, D. Joshua Liao

**Affiliations:** 1 Guangxi Key Laboratory of Livestock Genetic Improvement, Guangxi Institute of Animal Sciences, Nanning, Guangxi, P. R. China; 2 Hebei Research Institute for Family Planning, Shijiazhang, Hebei, P. R. China; 3 Hormel Institute, University of Minnesota, Austin, Minnesota, United States of America; Peiking University Third Hospital, CHINA

## Abstract

The maternal-to-embryonic transition (MET) is a complex process that occurs during early mammalian embryogenesis and is characterized by activation of the zygotic genome, initiation of embryonic transcription, and replacement of maternal mRNA with embryonic mRNA. The objective of this study was to reveal the temporal expression and localization patterns of PTTG1 during early porcine embryonic development and to establish a relationship between PTTG1 and the MET. To achieve this goal, reverse transcription-polymerase chain reaction (RT-PCR) was performed to clone porcine PTTG1. Subsequently, germinal vesicle (GV)- and metaphase II (MII)-stage oocytes, zygotes, 2-, 4-, and 8-cell-stage embryos, morulas, and blastocysts were produced *in vitro* and their gene expression was analyzed. The results revealed that the coding sequence of porcine PTTG1 is 609-bp in length and that it encodes a 202-aa polypeptide. Using qRT-PCR, PTTG1 mRNA expression was observed to be maintained at high levels in GV- and MII-stage oocytes. The transcript levels in oocytes were also significantly higher than those in embryos from the zygote to blastocyst stages. Immunohistochemical analyses revealed that porcine PTTG1 was primarily localized to the cytoplasm and partially localized to the nucleus. Furthermore, the PTTG1 protein levels in MII-stage oocytes and zygotes were significantly higher than those in embryos from the 2-cell to blastocyst stage. After fertilization, the level of this protein began to decrease gradually until the blastocyst stage. The results of our study suggest that porcine PTTG1 is a new candidate maternal effect gene (MEG) that may participate in the processes of oocyte maturation and zygotic genome activation during porcine embryogenesis.

## Introduction

The maternal-to-embryonic transition (MET) is a complex process that occurs during embryogenesis. At the onset of oocyte maturation, maternal factors accumulate in the ooplasm of eggs. Before oocyte maturation is completed, during the second meiotic division, a large number of maternal proteins and mRNAs are produced and stored in oocytes, and transcription and translation of these mRNAs pause. Once an oocyte has completed the second meiotic division or is activated by fertilization, transcription and translation of these mRNAs resume, sustaining the development of the early embryo [1]. Before the maternal reserves are exhausted, new mRNAs are produced by activation of zygotic genome transcription to ensure proper embryonic development. Subsequently, embryonic development is controlled by the zygotic nucleus [2]. During the MET, if embryonic genome activation is not completed, then new mRNAs cannot be synthetized, and embryonic development is blocked [3]. Therefore, the MET is causally linked to the developmental block of early mammalian embryogenesis [4,5]. Maternal effect genes (MEGs) in oocytes also play important roles in early embryos during the MET. Among these genes, those that activate embryonic genome transcription control early embryonic development to ensure that it follows a normal course [4]. Therefore, understanding the mechanisms and factors of the MET is important for improving the methods and conditions of not only the *in vitro* production of embryos [6,7] but also the generation of mammalian non-transgenic cloned embryos/offspring [8–11] and transgenic cloned embryos/offspring [12–15] using different approaches to somatic cell nuclear transfer.

Some MEGs, such as maternal antigens that embryos require, T-cell leukemia/lymphoma 1a, nucleophosmin/nucleoplasmin 2 and Filia, have been previously identified in mammals using various gene knockout approaches [16–19]. These MEGs share some common characteristics of zygotic genome activation (ZGA) and transcription initiation in embryos; however, their products have little effect on oocyte development and fertilization [20]. Although a few mammalian MEGs have been identified to date, there is limited information available regarding their molecular mechanisms during the MET.

Pituitary tumor transforming gene 1 (PTTG1), which encodes the mammalian protein securin, was originally isolated from rat pituitary tumor cells and was then identified as a member of the securin proteins family [21,22]. PTTG1 is a multifunctional protein that plays important roles in mitosis, cell transformation, DNA repair and transcriptional regulation [21,23–26]. In humans, PTTG1 expression is relatively high in the testis, thymus and placenta and very low or undetectable in the spleen, prostate, ovary, peripheral blood leukocytes, heart, brain, liver, skeletal muscle, kidney and pancreas [27,28]. However, it is expressed at very high levels in a variety of human endocrine-related neoplasms, such as pituitary, thyroid, ovarian and uterine tumors, and in some cancers derived from the central nervous, respiratory and gastrointestinal systems [22,29]. Some studies have suggested that PTTG1 might be a new candidate MEG in mammals because it plays roles in regulating oocyte maturation and in normal early embryonic development [30,31]. These studies have shown that PTTG1 expression gradually increases from the immature oocyte stage to the zygote stage and that elevated PTTG1 expression in blastocysts results in pregnancy failure after embryo transfer, suggesting that PTTG1 may play an important role in early mammalian embryogenesis.

The objective of this study was to clone porcine PTTG1 and determine its temporal expression and localization during early porcine embryo development to elucidate its role in the MET. We also assessed whether an association exists between PTTG1 mRNA and protein levels during early embryonic development.

## Materials and Methods

### Animal ethics

All animal samples used in our experiment were obtained from a slaughterhouse. All procedures involving animals and their care were performed according to the NIH guidelines (NIH Pub. No. 85–23, revised 1996) and were approved by Guangxi Institute of Animal Sciences.

### PTTG1 cDNA cloning

Ovaries from a 12-month-old Luchuan pig were obtained from a local slaughterhouse (Nanning Xuefeng Food Co., Ltd., Nanning, China) and frozen in liquid nitrogen. Total RNA was extracted using TRIzol reagent (Tiangen) and then stored at -80°C. The RNA was reverse transcribed (RT) into first-strand cDNA using M-MLV Reverse Transcriptase (Promega). To amplify the coding sequence of the PTTG1 gene in Luchuan pig, two cloning primers were designed based on the conserved region of the predicted *Sus scrofa* PTTG1 mRNA. The primer sequences were as follows: 5’-ATGTCTACTCTGATCTTTGTTGATA-3’ (P1, forward primer) and 5’-TTAAATATGTAAGTCATAGCAAACA-3’ (P2, reverse primer). Polymerase chain reaction (PCR) was performed in a 20-μL reaction volume containing 10 μL of 2× Taq PCR Master Mix, 1 μL of each primer (10 μmol L^−1^), 1 μL of cDNA template and 7 μL of H_2_O. PCR was initiated with denaturation at 94°C for 5 min, followed by 35 cycles of 30 s at 94°C, 30 s at 60°C and 60 s at 72°C, and a final extension for 10 min at 72°C. The PCR products were separated by 1.2% agarose gel electrophoresis. The fragment of interest was excised from the gel and purified using an agarose gel DNA fragment recovery kit (Tiangen). Then, it was cloned into a pMD-18T vector (TaKaRa). Finally, the plasmid was propagated and sequenced by FastBac (Lifei, Shanghai, China).

### Preparation of porcine early embryos

#### *In vitro* maturation

Porcine ovaries were collected from a local slaughterhouse and washed with maturation medium. Oocytes were then aspirated from 3-8-mm follicles. Oocytes with compact cumulus cells were matured *in vitro* in TCM199 medium (Gibco^TM^ Invitrogen Co.) supplemented with 10 IU/mL hCG and 10 IU/mL eCG at 39°C with 5% CO_2_ for 22 h. The oocytes were then transferred into another maturation medium without hormone supplementation and incubated for 18–20 h. Cumulus cells on cumulus-oocyte complexes (COCs) were stripped off after oocyte maturation. Matured oocytes with polar bodies were selected for *in vitro* fertilization.

#### *In vitro* fertilization

Six milliliters of mTBM was transferred to a centrifuge tube containing 0.8 mL fresh porcine sperm, mixed and then centrifuged at 850 rpm for 5 min. After the supernatant was removed, 0.5 mL of the remaining solution was transferred to a new centrifuge tube, and 5 mL porcine fertilization medium (PF) was added. The mixture was centrifuged at 850 rpm for 3 min. After the supernatant was removed, 0.3 mL of the remaining solution was transferred to a 1.5 mL Eppendorf tube (EP) tube for fertilization. Matured oocytes were washed sequentially in porcine zygote medium 3 (PZM3) and PF and then transferred to a four-well plate containing PF. Treated sperm were also transferred to the plate, followed by culturing at 39°C and 5% CO_2_ for 3 h. Fertilized oocytes were washed in PZM3 to remove excess sperm and re-cultured in PZM3 at 39°C and 5% CO_2_ for 4–168 h. Two-cell, 4-cell, and 8-cell embryos, morulas, and blastocysts were collected at 4, 48, 96, 120 and 168 h after culture *in vitro*, respectively. These embryos at different stages were used for subsequent quantitative real-time RT-PCR (qRT-PCR) and immunohistochemical analyses.

### Quantitative real-time RT-PCR

The PTTG1 mRNA levels in oocytes and embryos were determined by qRT-PCR with a forward primer (5’-CCCTCAAACAGAAACAGACA-3’) and reverse primer (5’-GGATAGTTGTCATCTGAGGC-3’). These levels were calculated relative to 18S rRNA using the **2**^-ΔΔCt^ method. qRT-PCR was performed at least 14 times for each of the different stages of oocytes and embryos.

### Immunohistochemistry

PTTG1 protein expression in oocytes and embryos was determined by immunohistochemistry. To remove residual blood, the oocytes and embryos were washed three times with DPBS containing 0.01% Triton X-100 and 0.3% bovine serum albumin (BSA; Sigma). They were then fixed in 4% (w/v) paraformaldehyde for 30 min and washed three times for 5 min each with DPBS containing 0.3% BSA. Next, the oocytes and embryos were incubated in 1% Triton X-100 for 15 min and washed three times for 5 min each with DPBS containing 0.3% BSA. Subsequently, they were incubated in PBS containing 1% BSA for 1 h and rinsed three times with DPBS containing 0.01% Triton X-100 and 0.3% BSA. The oocytes and embryos were then incubated with a primary antibody (rabbit monoclonal anti-PTTG1, diluted 1:100 in 0.3% BSA; GeneTex, San Antonio, Texas, USA) at 4°C overnight. The next day, they were incubated at 37°C for 20 min and washed three times for 5 min each with DPBS containing 0.01% Triton X-100 and 0.3% BSA. Then, a second anti-PTTG1 primary antibody (FITC-labeled goat anti-rabbit IgG, diluted 1:200; GeneTex, San Antonio, Texas, USA) was applied. Subsequently, the oocytes and embryos were incubated with a second antibody for 90 min at room temperature and stained with 10 μg/ml propidium iodide (PI; Sigma) for 5 min. After staining, the oocytes and embryos were rinsed with DPBS containing 0.01% Triton X-100 and 0.3% BSA and transferred onto a special slide coated with an anti-quenching agent. The cover slip was sealed using paraffin and vaseline. Green and red fluorescence signals from the oocytes and embryos were observed at excitation wavelength of 488 nm and 536 nm, respectively, using a laser scanning confocal microscope (Leica TCS SP5, Germany).

### Statistical analysis

All data are expressed as the mean±standard deviation (SD). Statistical analyses were performed using ANOVA with SPSS 17.0 software. Comparisons among groups were performed using Duncan’s multiple comparisons test. A *P* value<0.05 was considered significant.

## Results

### cDNA cloning and PTTG1 mRNA sequence analysis

**Fig 1:**
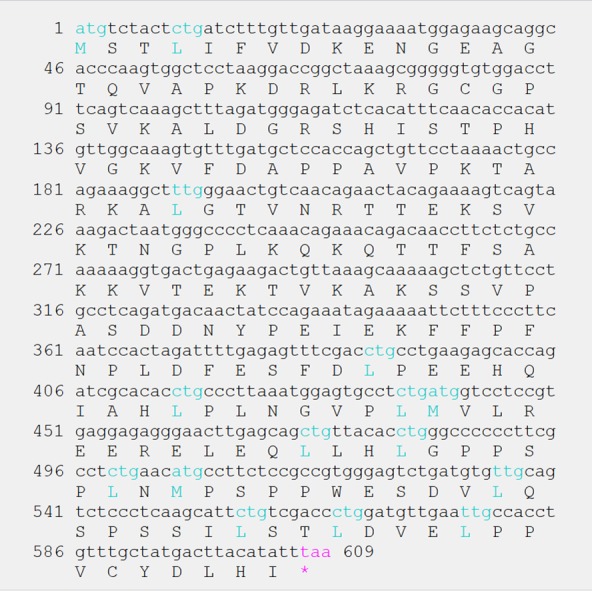
The coding sequence and amino acid sequence of PTTG1 cloned from a Luchuan pig (length: 202 aa), with start codons and stop codons in bold.

**Fig 2:**
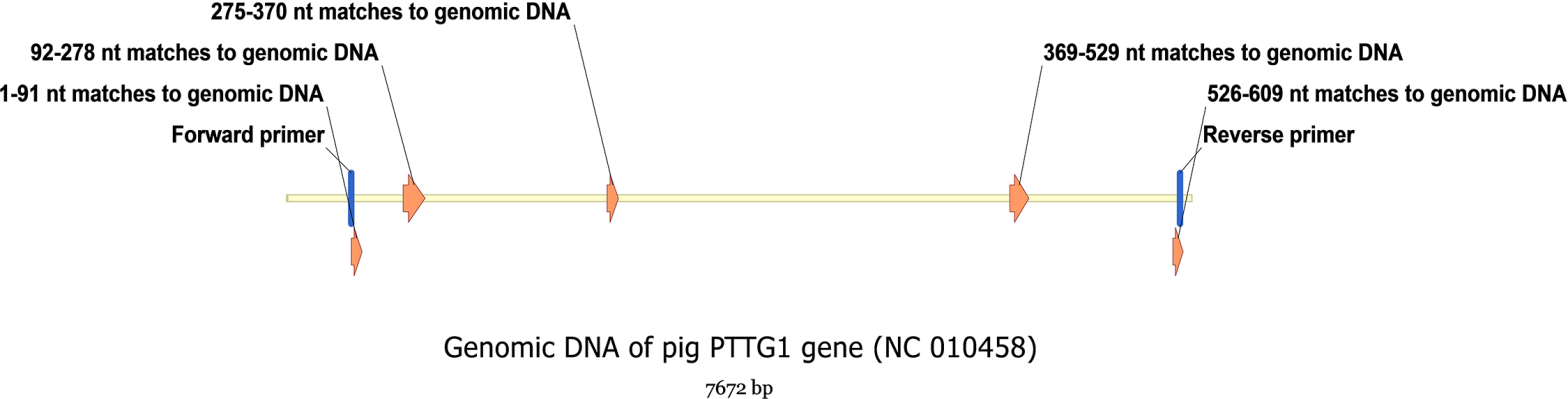
Sequence mapping of the cloned fragment to porcine PTTG1 genomic DNA from NCBI database, showing that the porcine PTTG1 gene contains five exons.

A cDNA fragment was amplified from the RNA sample using the primers P1 and P2. Sequencing of the fragment resulted in a 609-bp sequence encoding a 202-amino acid (aa) open reading frame (Fig 1). Nucleotide sequence alignment revealed that it had differing degrees of similarity with cattle (90.15%), human (87.52%) and mouse PTTG1 mRNA (74.38%). High levels of similarity (up to 71.29%) were also observed between the protein sequences of the fragment and those of cattle, human, mouse and other species. BLAST analysis of the genomic DNA sequence (NC_010458) indicated that the target sequence spanned from the 538 bp position (start codon) to the 7595 bp position (stop codon) and potentially contained 5 coding exons (Fig 2). These results indicated that the obtained fragment included a portion of the coding region of the porcine PTTG1 gene.

### PTTG1 expression in porcine oocytes and embryos

**Fig 3:**
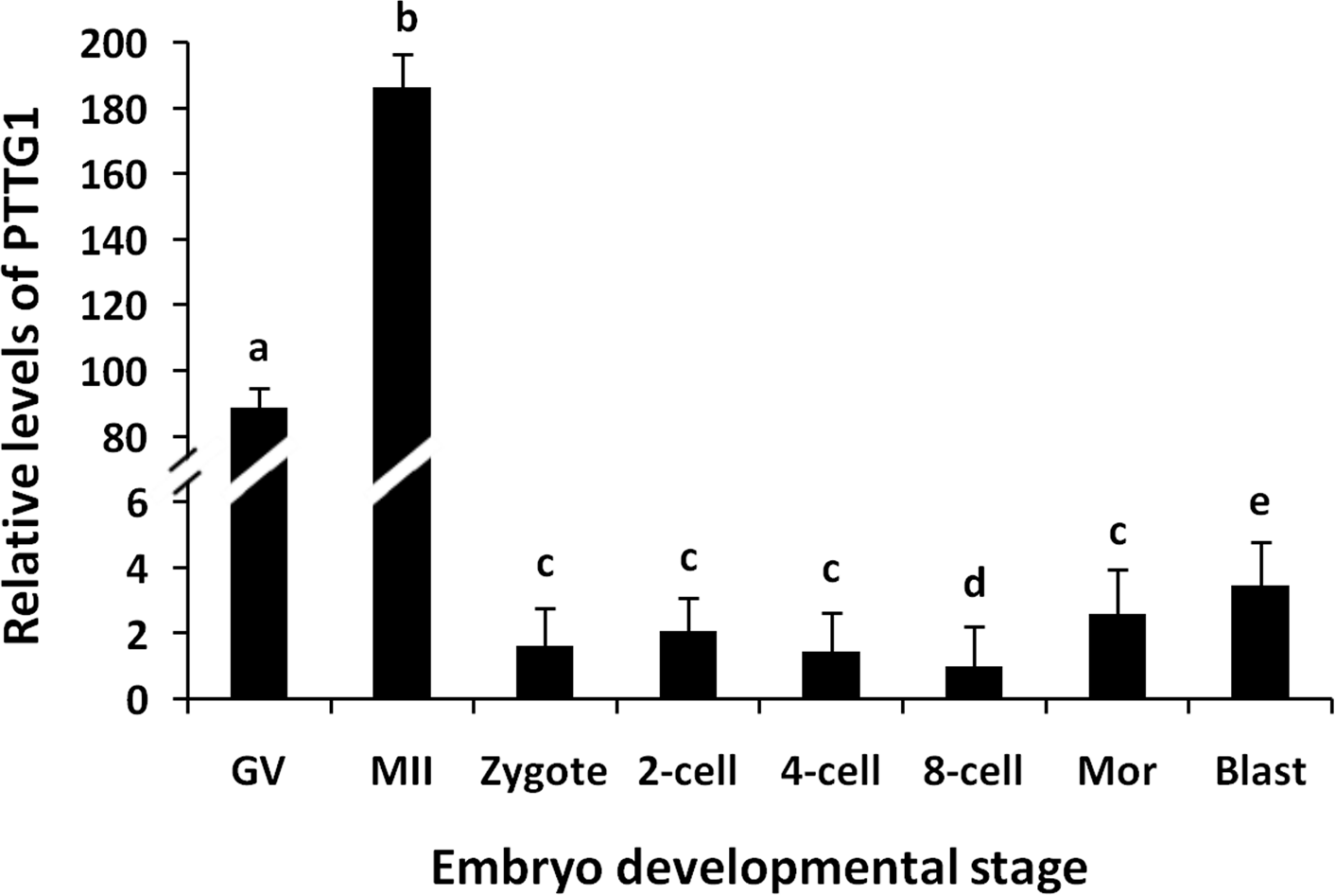
Porcine PTTG1 mRNA expression in oocytes and embryos at different developmental stages. GV, germinal vesicle oocytes; MII, metaphase II oocytes; Mor, morula; Blast, blastocyst. Bars with different letters (a, b, c, d, and e) indicate significant differences at P<0.05.

qRT-PCR was then performed using 18S rRNA for calibration. The results indicate that PTTG1 mRNA was expressed in porcine oocytes and embryos from the zygote to blastocyst stages, with high levels of expression in germinal vesicle (GV)- and metaphase II (MII)-stage oocytes. The transcript levels in these oocytes were significantly higher than those in the embryos at the zygote to blastocyst stages. In addition, low but consistently detectable PTTG1 mRNA expression was observed during all stages of embryonic development, with a gradual decrease from the 2- to 8-cell stage. However, its expression increased gradually in the embryos from the 8-cell to the blastocyst stages, with the highest level detected in blastocysts relative to embryos in the zygote to morula stages (Fig 3).

### Expression and localization of the PTTG1 protein in oocytes and embryos

**Fig 4:**
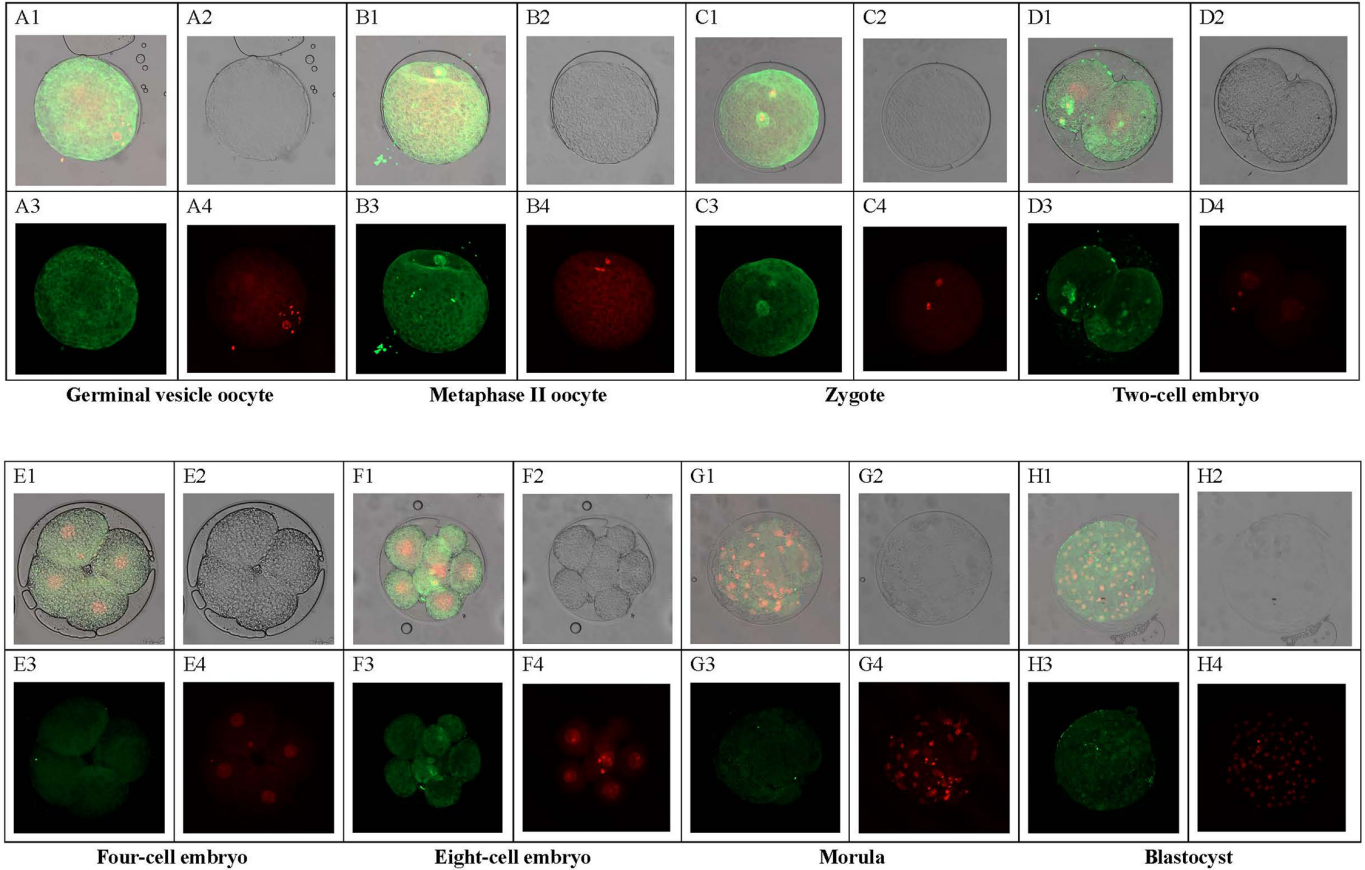
Immunohistochemical staining of PTTG1 in porcine oocytes and embryos (magnification, 400X). A, germinal vesicle oocyte; B, metaphase II oocyte; C, zygote; D, two-cell embryo; E, four-cell embryo; F, eight-cell embryo; G, morula; and H, blastocyst. Top left corner (A1, B1, C1, D1, E1, F1, G1, and H1), a composite image from the lower left corner and lower right corner; top right corner (A2, B2, C2, D2, E2, F2, G2, and H2), oocytes and embryos were not dyed with any coloring agent; lower left corner (A3, B3, C3, D3, E3, F3, G3, and H3), oocytes and embryos were dyed with FITC-labeled goat anti-rabbit IgG, an antibody specific for PTTG1; and lower right corner (A4, B4, C4, D4, E4, F4, G4, and H4), nuclei of oocytes and embryos were dyed with propidium iodide.

**Fig 5:**
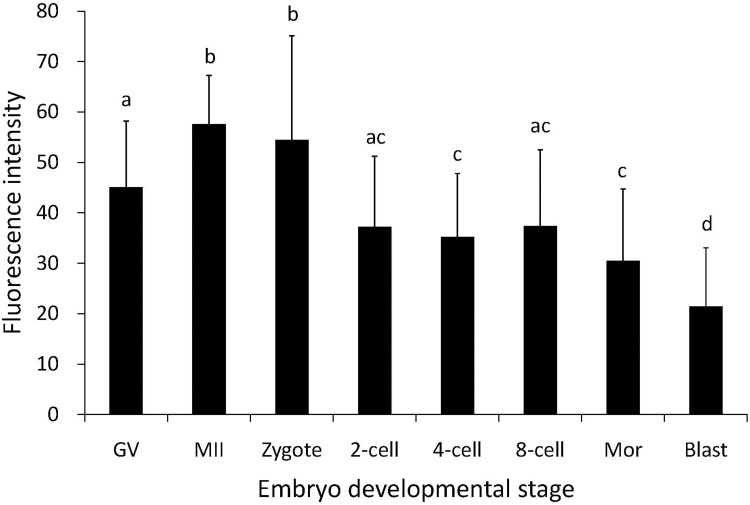
Comparison of the fluorescence intensities of porcine oocytes and embryos at different developmental stages. GV, germinal vesicle oocytes; MII, metaphase II oocytes; Mor, morula; Blast, blastocyst. Bars with different letters (a, b, c, and d) indicate significant differences at P<0.05.

Immunohistochemical staining with a PTTG1 antibody that recognizes porcine PTTG1 revealed that the PTTG1 protein was localized to both the nuclei and cytoplasm of oocytes and embryos, with homogeneously distributed fluorescence in the cytoplasm of oocytes and embryonic cells. The staining of the nuclei and cytoplasm of oocytes (GV and MII stages) and zygotes was more intense than that of the nuclei and cytoplasm of whole embryos from the 2-cell to blastocyst stages (Fig 4). We converted the fluorescence intensities to numerical values to quantify the protein levels. The results showed that the protein levels were significantly higher in the MII-stage oocytes and zygotes than in the embryos from the 2-cell to blastocyst stages. In addition, the protein level gradually increased with oocyte maturation and peaked at the MII stage of oocytes. After fertilization, the protein level began to decrease gradually until the blastocyst stage (Fig 5). These data indicate that PTTG1 protein expression may play a role in porcine oocyte maturation.

## Discussion

Many studies have shown that PTTG1 plays important roles in mitosis, cell transformation, DNA repair and transcriptional regulation. Although it has also been demonstrated to be a novel oncogene in humans [28], its roles in mammals, particularly its physiological activities, have not been sufficiently examined. Therefore, we investigated the temporal expression and localization of PTTG1 in porcine oocytes and embryos after the cloning of this gene from a pig.

Human PTTG1 is located on chromosome 5, and it encodes a 202-aa, 23-kD protein [32], whereas rat PTTG1 mRNA is 974 nt in length and encodes a 199-aa protein [21], and cattle PTTG1 mRNA is 1464-bp in length and encodes a 202-aa protein [33]. Due to the lack of sequencing information for porcine PTTG1, we cloned a portion of its mRNA sequence in this study. Our results showed that the open reading frame of the porcine PTTG1 mRNA is 609 nt long and encodes a 202-aa protein, as in humans and cattle. Sequence alignment revealed high similarities in the deduced PTTG1 aa sequences among species including mouse, pig, cattle, and human, suggesting that PTTG1 is relatively evolutionarily conserved among mammals.

Many studies have demonstrated that early embryonic development does not depend on the cell nucleus but rather that it relies on maternal factors stored in oocytes [20,34,35]. These maternal factors regulate the differentiation of oocytes into embryos during early embryonic development and regulate ZGA. MEGs play important roles during the accumulation of maternal factors. Disruption of MEG expression results in defective embryogenesis. Therefore, a close correlation exists between MEGs and ZGA. During the MET, embryonic genome transcription is inactive before initiation of ZGA, and the time of initiation varies among species. In mice, ZGA occurs as early as the S/G2 phase in the paternal pronucleus of the 1-cell embryo and strengthens at the 2-cell embryo stage [36–38]. Moreover, ZGA is initiated at the 4-cell stage in pigs [39], at the 8-cell stage in sheep [40], and at the 4- and 8-cell stages in humans [41], whereas it is delayed until the 8-16-cell stage in cows [42]. Our results showed that the PTTG1 mRNA and protein levels were the lowest at the 8- and 4-cell stages, respectively, and that the protein expression pattern is similar to that reported for embryonic development from the zygote to blastocyst stages in cows, although the lowest PTTG1 protein level in cows has been observed at the 8-cell stage [42]. Based on these data, we conclude that ZGA occurs at the 4- to 8-cell stage in pigs.

PTTG1 is a mammalian homolog of *Xenopus* securin. It ensures for equal distribution of genetic material through mitosis [22,43] and mediates transcriptional activation [44]. Thus, it likely plays an important role during embryogenesis. El-Sayed et al. have shown that blastocysts containing a high level of PTTG1 transcripts do not lead to pregnancy after being transferred to recipients [31]. In bovine oocytes, the PTTG1 mRNA level is higher in dominance phase oocytes than in their growth phase counterparts, which may lead to a reduced developmental capacity of enclosed oocytes [30], whereas the PTTG1 mRNA level in mouse zygotes is 39% higher than that in mouse oocytes, suggesting that PTTG1 may play a role in ZGA [45]. We observed that the PTTG1 mRNA levels were significantly higher in oocytes than in embryos from the zygote to blastocyst stages and that the protein levels were significantly higher in MII-stage oocytes and zygotes than in cells at other embryonic stages. Moreover, the high PTTG1 protein level was sustained until the 8-cell embryo stage. We also found that the abundant PTTG1 transcripts in oocytes promoted porcine oocyte maturation. Taken together, these results suggest that PTTG1 may play an important role in ZGA.

We observed that the PTTG1 protein is primarily localized to the cytoplasm and only partially localized to the nucleus, in accordance with the observations of Dominguez and Chien [27,46]. This predominantly cytoplasmic localization is inconsistent with the conclusions of many studies suggesting that PTTG1 is an important transcriptional activator [46,47] and that its transcriptional activity is activated by epidermal growth factor [48]. In some germ cell-derived tumors, the localization of the PTTG1 protein remains uncertain, likely due to genomic instability. Pierconti et al. have shown that PTTG1 is primarily localized to the nucleus in central areas of seminomas but that it is more intense in the cytoplasm in the peripheral areas of tumors [49]. Moreover, in early porcine embryos, increased PTTG1 mRNA levels in oocytes and embryos have not been found to be associated with increased protein levels, which is another discrepancy with our data. GV-stage oocytes most likely need to accumulate large amounts of mRNAs to achieve the MET and to participate in ZGA.

In conclusion, we have cloned the coding region of PTTG1 from a Luchuan pig and have demonstrated the temporal expression and localization of PTTG1 in early porcine embryogenesis for the first time. In addition, the expression data have revealed several likely physiological functions of PTTG1 and the initiation period of ZGA in porcine oocytes and embryos. These findings suggest that pig PTTG1 may be a new candidate MEG that participates in oocyte maturation and ZGA during embryogenesis.
